# Rapid Identification of Aldose Reductase Inhibitory Compounds from *Perilla frutescens*

**DOI:** 10.1155/2013/679463

**Published:** 2013-11-06

**Authors:** Ji Hun Paek, Kuk Hyun Shin, Young-Hee Kang, Jae-Yong Lee, Soon Sung Lim

**Affiliations:** ^1^Department of Food Science and Nutrition, Hallym University, 1 Hallymdaehak-gil, Chuncheon, Gangwon-do 200-702, Republic of Korea; ^2^Korea Institute of Science and Technology Information, Seoul 130-741, Republic of Korea; ^3^Department of Center for Aging and Health Care, Hallym University, 1 Hallymdaehak-gil, Chuncheon, Gangwon-do 200-702, Republic of Korea; ^4^Department of Biochemistry, School of Medicine, Hallym University, 1 Hallymdaehak-gil, Chuncheon, Gangwon-do 200-702, Republic of Korea; ^5^Institute of Natural Medicine, Hallym University, 1 Hallymdaehak-gil, Chuncheon, Gangwon-do 200-702, Republic of Korea

## Abstract

The ethyl acetate (EtOAc) soluble fraction of methanol extracts of *Perilla frutescens* (*P. frutescens*) inhibits aldose reductase (AR), the key enzyme in the polyol pathway. Our investigation of inhibitory compounds from the EtOAc soluble fraction of *P. frutescens* was followed by identification of the inhibitory compounds by a combination of HPLC microfractionation and a 96-well enzyme assay. This allowed the biological activities to be efficiently matched with selected HPLC peaks. Structural analyses of the active compounds were performed by LC-MS^n^. The main AR inhibiting compounds were tentatively identified as chlorogenic acid and rosmarinic acid by LC-MS^n^. A two-step high speed counter current chromatography (HSCCC) isolation method was developed with a solvent system of n-hexane-ethyl acetate-methanol-water at 1.5 : 5 : 1 : 5, v/v and 3 : 7 : 5 : 5, v/v. The chemical structures of the isolated compounds were determined by ^1^H- and ^13^C-nuclear magnetic resonance spectrometry (NMR). The main compounds inhibiting AR in the EtOAc fraction of methanol extracts of *P. frutescens* were identified as chlorogenic acid (**2**) (IC_50_ = 3.16 **μ**M), rosmarinic acid (**4**) (IC_50_ = 2.77 **μ**M), luteolin (**5**) (IC_50_ = 6.34 **μ**M), and methyl rosmarinic acid (**6**) (IC_50_ = 4.03 **μ**M).

## 1. Introduction

Long-term secondary diabetic complications are the main cause of morbidity and mortality in diabetic patients [1]. Recent human genetic and biochemical data link polymorphisms of the aldose reductase (AR) gene (technically called the AR2 gene) and elevated tissue levels of AR with strongly altered risks for diabetic complications [2]. Due to its proposed involvement in diabetic complications, ALR2 has been a drug target in the clinical management of diabetes [3]. Numerous clinical trials and experimental animal studies have shown that early intervention is required to achieve maximal reduction in the onset and severity of diabetic retinopathy and cataracts [4]. Natural or synthetic compounds such as flavonoids, benzopyrans, spirohydantoins, and quinones inhibit the enzyme with various degrees of activity and specificity [5, 6].

*Perilla frutescens* (*P. frutescens*) is an annual short-day plant belonging to the family Labiatae [7]. It has long been used as traditional folk medicine for anxiety, tumor, cough, bacterial and fungal infections, allergy, intoxication, and some intestinal disorders [8–11]. *P. frutescens *possesses anti-inflammatory, antitumor, and antiallergic activities [12]. Previous chemical studies on leaves and seeds of *P. frutescen*s have reported the presence of sterols: b-sitosterol, stigmasterol, and campesterol; terpenoids: ursolic and acid, oleanolic acid, and tormentic acid; anthocyanin: shisonin; flavonoids: apigenin, luteolin, and scutellarein; and phenolic acids: rosmarinic acid, caffeic acid, and ferulic acid [13–16]. Therefore, we investigated the inhibitory effect of the dried leaves of *P. frutescens* on AR to evaluate its potential in treating diabetic complications. The goal of this study was to identify the active constituents of *P. frutescens* by enzyme assay-guided HPLC microfractionation and to improve our understanding of how the active compound of* P. frutescens* acts against rAR.

## 2. Materials and Methods

### 2.1. Apparatus and Reagents

 DL-Glyceraldehyde, the reduced form of nicotinamide adenine dinucleotide phosphate (NADPH), sodium phosphate, and quercetin used in this study, was purchased from Sigma-Aldrich (St. Louis, MO, USA). All other chemicals and reagents used were of analytical grade.

### 2.2. Plant Materials

*P. frutescens *was purchased from a local market in Chuncheon, Rebublic of Korea. A voucher sample (RIC-2012-5) has been deposited at the Center for Efficacy Assessment and Development of Functional Foods and Drugs, Hallym University, Chuncheon. The specimens were authenticated by Emeritus Professor H. J. Chi, Seoul National University, Rebublic of Korea.

### 2.3. Extraction

Dried leaves of *P. frutescens *(2 kg) were extracted 3 times with 99.5% methanol for 5 h. The solvent was evaporated under reduced pressure below 45°C to give a methanol extract (yield: 11.68%). The extract was suspended in distilled water and partitioned with *n*-hexane (*n*-Hex), methylene chloride (CH_2_Cl_3_), ethyl acetate (EtOAc), *n*-butanol (*n*-BuOH), and H_2_O to yield *n*-Hex (40.83 g), EtOAc (25.20 g), CH_2_Cl_3_ (22.24 g), *n*-BuOH (116.88 g), and H_2_O fractions (27.42 g). In total, 6 fractions were obtained. A small amount of each fraction was dissolved in dimethyl sulfoxide (DMSO) and assayed for AR inhibitory activity.

### 2.4. HPLC Microfractionation

**Figure 1:**
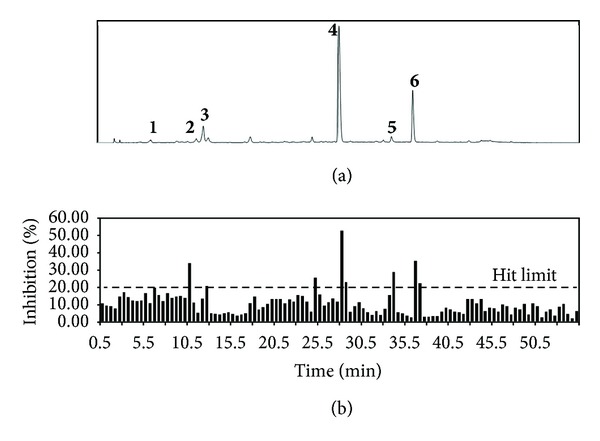
HPLC chromatogram (a) and active profile (b) of the HPLC microfractionated of EtOAc soluble fraction of *P. frutescens* for rAR inhibition in 96-well plate.

An automated fraction collector (Foxy 200; ISCO, Lincoln, NE, USA) coupled with HPLC equipment (Thermo Electron Spectra HPLC system; Thermo Separation Products, San Jose, CA, USA) was used to separate and collect compounds from extracts directly into 96-well plates (Nunc, Roskilde, Denmark) at 0.4 mL/well (Figure 1). HPLC separation was achieved with Agilent Eclips XDB-C18 columns (150 × 4.6 mm, 5 *μ*m, Agilent Tech., Palo Alto, CA, USA). The mobile phase, consisting of acetonitrile (ACN) and 0.1% aqueous trifluoroacetic acid (TFA), was used at a flow rate of 0.8 mL/min. The gradient elution program was 7.5–26.5% B (0–20 min), 26.5% B (20–30 min), 26.5–36% B (30–40 min), and 36–100% B (40–45 min). Injection volume was 10 *μ*L at sample concentration 10 mg/mL and detection wavelength 280 nm. After collection, all fractions were evaporated to dryness using an EZ-2 plus evaporator (Gene Vac Ltd., Ipswich, UK).

### 2.5. Validation of HPLC Microfractionation Assays

The effect on rAR inhibitory activity (%) was calculated as the change in absorbance in a sample well versus the change in absorbance in a blank well (average, *n* = 12). Spontaneous hydrolysis was subtracted from the reaction rate. The hit limit was set at 3 standard deviations (SD) from the minimum AR inhibitory activity. *Z*′-factor, signal-to-background ratio (*S*/*B*) [17], and signal-to-noise ratio(*S*/*N*) [18] were used to evaluate the quality of the test: *Z*′ = 1[(3 × SD_*s*_ + 3 × SD_*b*_)/|*X*_*s*_ *X*_*b*_|], *S*/*B* = *X*_*s*_/*X*_*b*_, and *S*/*N* = (*X*_*s*_ *X*_*b*_)/(SD_*s*_^2^ + SD_*b*_^2^)^1/2^; *X*_*s*_ and SD_*s*_ are average and standard deviation of the signal, that is, absorption detected at the 8th measurement point in blank wells (*n* = 12) after adding the enzyme; *X*_*b*_ and SD_*b*_ are average and SD of the background, that is, absorption at the first measurement point in blank wells (*n* = 12) after adding the enzyme.

### 2.6. LC/DAD/ESI-MS^n^ Analysis

The EtOAc extracts were analyzed by HPLC-PDA. In order to acquire chromatograms and UV spectra, we used the Finnigan Surveyor HPLC system (Thermo Electron, San Jose, CA, USA), which comprised a PDA plus detector, autosampler plus, a column compartment, and MS pump plus. The samples were separated on an Eclipse SB-C18 Rapid Resolution column (150 × 4.6 mm, 3.5 *μ*m, Agilent) with a C18 guard column (7.5 × 4.6 mm, i.d. 3.5 *μ*m, Alltech) using 0.1% trifluoroacetic acid (TFA) in a water (A) and acetonitrile (ACN) gradient system. The gradient elution program was 7.5–55% B (0–40 min), 55–100% B (40–50 min), and 100–100% B (50–60 min). The UV detector was set at 280 nm with full spectral scanning from 200 to 600 nm. Chromatography was performed at room temperature with a flow rate of 0.7 mL/min, and the injection volume was 10 *μ*L. Identification of the EtOAc extract was carried out by UV-DAD and ESI-MS. LC-ESI-MS analysis was performed using an ion trap mass spectrometer (Finnigan LCQ Advantage Max, Thermo) equipped with an electrospray ionization source as interface, coupled to a Finnigan Surveyor HPLC system (Thermo Electron Corporation). Ultrapure helium (He) was used as the collision gas, and pure nitrogen (N_2_) was used as the nebulizing gas. The mass spectrometer conditions were optimized using flow injection analysis of crude extract without HPLC column and were as follows: source voltage 4.8 kV, capillary voltage 3.0 V, sheath gas flow rate 8.0 mL/min; and capillary temperature 275.0°C; the first event was a full (200 to 700) MS scan (MS^1^); during the second event, the main ion recorded was isolated and selectively fragmented in the ion trap (MS^2^); collision energy for fragmentation 40 eV. ^1^H and ^13^C NMR spectra of the isolated pure compounds were recorded with a Bruker AV 600 instrument, using MeOH-d4 or DMSO-d6 as a solvent.

### 2.7. Measurement of Partition Coefficient and Settling Time for HSCCC

Two-phase solvent systems were tested by changing the volume of the solvent to obtain the optimum composition to yield suitable partition coefficient (*K* values). The *K* values were determined as described [19]. Briefly, the composition of a two-phase solvent system was selected according to the *K* of the target compounds of crude extract. Approximately 25 mg of the crude extract was weighed in a 20 mL test tube to which 5 mL of each phase of the preequilibrated two-phase solvent system was added. After the tube was shaken vigorously, the solution was quickly separated for a moment. Then, the upper and lower phases were analyzed by HPLC to obtain the *K* value of the target compound. The *K* value was expressed as the peak area of the target compound in the upper phase divided by that of the lower phase. Settling time, which is closely correlated to retention of the stationary phase, was expressed as the time needed to form a clear layer between phases when each phase (1 : 1, v/v) was mixed.

### 2.8. Preparation of Two-Phase Solvent System and Sample Solution for HSCCC

The two-phase solvent composed of n-hexane-ethyl acetate-methanol-water HEMWat, 1.5 : 5 : 1 : 5, v/v, and 3 : 7 : 5 : 5, v/v, was used for HSCCC separations. Each component of the solvent system was added to a separate funnel and thoroughly equilibrated at room temperature. Two phases were separated and degassed by sonication for 30 min before use. The sample solutions were prepared by dissolving 4.0 g of the crude extract in the mixture of upper and lower phases (1 : 1, v/v) of the solvent system used for HSCCC separation.

### 2.9. HSCCC Separation

The multilayer coil column was entirely filled with the upper organic phase (stationary phase) at a flow rate of 10.0 mL/min. The lower phase was then pumped into the head of the inlet column at a flow rate of 5.0 mL/min, while the apparatus was run at a revolution speed of 400 rpm. After hydrodynamic equilibrium was established, as indicated by a clear mobile phase eluting at the tail outlet, the sample solution (4.0 g in 50 mL of each phase) was injected into the separation column through the injection valve. The effluent from the tail end of the column was continuously monitored by a connection to a coiled column with a UV detector at 280 nm. Each peak fraction was collected in 25 mL tubes according to the elution profile. After the separation was complete, stationary phase retention was measured by collecting the column contents; this was done by forcing them out of the column with pressurized nitrogen gas.

### 2.10. Assay for rAR Inhibitory Activity

Crude rAR was prepared as follows. Rat lenses were removed from closed male Sprague-Dawley rats weighing 250–280 g and frozen until required. The rat lens homogenate was prepared according to Hayman and Kinoshita with some modifications [20, 21]. A partially purified enzyme with a specific activity of 6.5 U/mg was routinely used to test enzyme inhibition. The partially purified material was separated into 1.0 mL aliquots and stored at 40°C. rAR activity was assayed spectrophotometrically by measuring the decrease in NADPH absorption at 340 nm over a 4 min period with dl-glyceraldehyde substrate. Each 1.0 mL cuvette contained equal units of the enzyme, 0.10 M sodium phosphate buffer (pH 6.2), and 0.3 mM NADPH, with or without 10 mM substrate and an inhibitor [22, 23]. The concentration of inhibitors yielding 50% inhibition (IC_50_) was calculated from the least-squares regression line of the logarithmic concentrations plotted against the residual activity.

## 3. Results

### 3.1. rAR Inhibitory Effects of the Crude Extracts

The purpose of this study was to identify a natural AR inhibitor from *P. frutescens *to be used in the treatment of diabetic complications. To identify the active compounds from *P. frutescens*, plant extracts were systematically divided into 6 fractions, which were then tested for AR inhibitory activity. The EtOAc fraction exhibited the strongest rAR inhibitory activity, with IC_50_ 1.92 *μ*g/mL (Table 1). The IC_50_ for quercetin, a well-known AR inhibitor used as the reference control in this study, was 2.29 *μ*g/mL. These results suggest that the EtOAc soluble fraction of *P. frutescens *contains abundant AR inhibitory compounds.

### 3.2. rAR Inhibitory Activities after HPLC Microfractionation

The EtOAc soluble fraction of *P. frutescens *inhibited rAR. Therefore, we sought to identify the rAR inhibitory compounds by combining HPLC microfractionation with a 96-well enzyme assay. This enabled the biological activities to be efficiently matched with specific HPLC peaks (Figure 1). Inhibition was linked to a substance that eluted at peaks **2** and **4**. The rAR inhibitory activity in the corresponding wells was 34.50 and 53.31%, respectively. Similar inhibition values at peaks **1** (20.46%), **3** (21.31%), **5** (29.46%), and **6** (22.94%) were close to the hit limit. Further efforts to identify and characterize this substance are ongoing. Dereplication assays using HPLC play an important role in the search for active compounds from plants, providing rapid access to information concerning both the activity and localization of the activity in complex plant matrices. The active compounds at peaks **2** and **4 **were tentatively identified as chlorogenic acid and rosmarinic acid by LC-ESI/MS^n^. The negative ion mass spectra of peak **2** show an ion at *m*/*z* 353 (M-H)^−^ and fragment ions at *m*/*z* 191 [quinic acid-H]^−^ and 179 [caffeic acid-H]^−^; these were compared to the elution order of caffeoylquinic acids reported in the literature [24]. Peak **2** was identified as chlorogenic acid. The positive ion mass spectra of peak **4** show an ion at *m*/*z* 360.85 [M]^+^ and fragment ions at *m*/*z* 342.93 [M-H_2_O]^+^, 180.94 [caffeic acid]^+^, and 163.1 [caffeic acid esters]^+^, which is consistent with the observation of *m*/*z* 163 in the positive ion mode, typical of caffeic acid esters [25, 26]. Peak **4** was identified as rosmarinic acid.

The quality of the assay was assessed using statistical parameters to ensure sufficient dynamic range and acceptable signal variability. The results were considered valid when *Z* = 0.5, *S*/*N* = 10, and *S*/*B* = 4. In general, the limiting value of 0.5 for the *Z*′-factor indicates an excellent assay [17]. The standard deviation in the blank wells (*n* = 12) was 7.39%, and thus the hit limit was set at 18.49%. Calculated statistical parameters for the assay were excellent (*Z*′ = 0.96, *S*/*N* = 12.12, and *S*/*B* = 13.46).

### 3.3. HSCCC Separation

**Figure 2:**
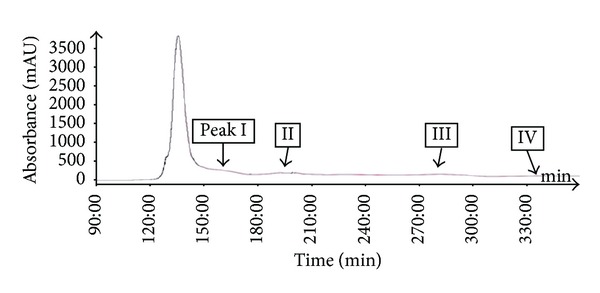
HSCCC separation of the EtOAc soluble fraction from the leaves of *P. frutescens*. Solvent system: n-hexane-ethyl acetate-methanol-water (1.5 : 5 : 1 : 5, v/v); flow-rate, 5.0 mL/min; revolution speed, 400 rpm; sample size, 4.0 g; injection volume, 50 mL; detection wavelength, 280 nm; stationary phase retention, 56%. Peaks I, II, III, and IV in the HSCCC chromatogram correspond to compounds **3**,** 1**,** 2**, and **4**, respectively.

**Figure 3:**
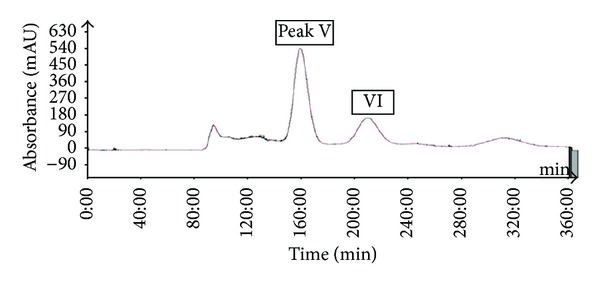
HSCCC chromatogram of *P. frutescens* after HSCCC separation. Solvent system: n-hexane-ethyl acetate-methanol-water (3 : 7 : 5 : 5, v/v); flow rate, 5.0 mL/min; revolution speed, 400 rpm; sample size, 1.2 g; injection volume, 50 mL; detection wavelength, 280 nm; stationary phase retention, 53%. Peaks V and VI in the HSCCC chromatogram correspond to compounds **6** and **5**, respectively.

**Figure 4:**
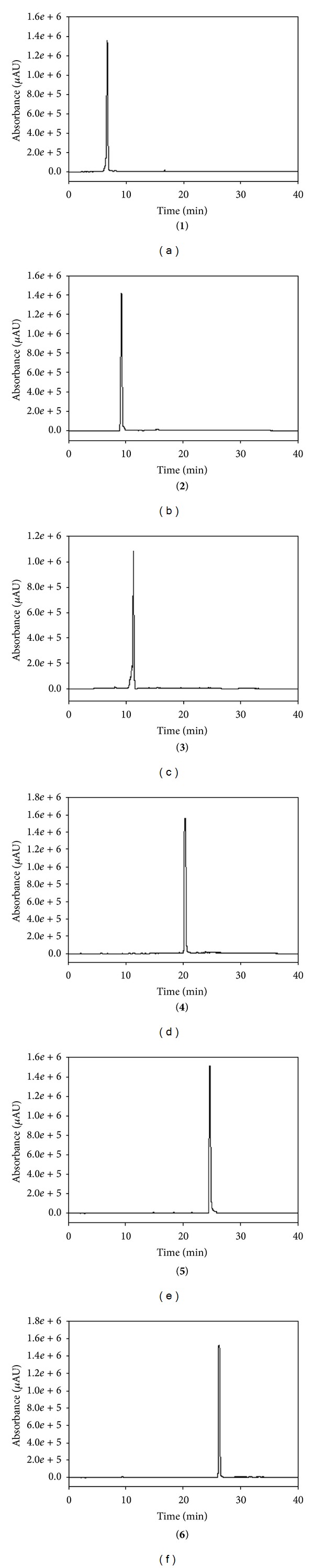
HPLC analysis of compounds **1**–**6** separated by HSCCC. Conditions: column, Eclipse SB-C18 Rapid Resolution column (150 × 4.6 mm, 5 *μ*m, Agilent); column temperature, 30°C; mobile phase, 0.1% TFA (solvent A) and acetonitrile (solvent B); HPLC analysis, linear gradient from 7.5 to 55% B (0–40 min). Flow rate, 0.7 mL/min; detection, photodiode array detector; injection volume, 10 *μ*L.

The most important step in the design of an HSCCC separation protocol is selection of the solvent system. Generally, the two-phase solvent system must satisfy the following requirements: (i) the settling time of the solvent system should ideally be shorter than 30 s to ensure satisfactory retention of the stationary phase, (ii) the partition coefficient (*K*) of the target compounds should lie within the range 0.5 ≤ *K* ≤ 2.5 for efficient separation, and (iii) the separation factor between the components (*α* = *K*2/*K*1, *K*2 > *K*1) should be greater than 1.5 [27, 28]. In this study, the *K* values of 6 compounds were determined by HPLC, as described in the Materials and Methods. The measured *K* values of each compound are summarized in Table 2. Based on the criteria for *K* values in the range of 0.5–2.5, one system was selected for further evaluation which was HEMWat (1.5 : 5 : 1 : 5, v/v). As presented in Table 2, using HEMWat solvent systems, the *K* values of compounds **1**–**4** were suitable, whereas those of compounds **5** and **6** were too great. Thus, HSCCC separation could not be performed using the single two-phase solvent. First, the 4.0 g quantity of EtOAc soluble fraction of *P. frutescens* was subjected to HSCCC using HEMWat solvent (1.5 : 5 : 1 : 5, v/v). The separation time was 360 min, and stationary phase retention was 56%. As shown in Figure 2, there are 4 isolated peaks I–IV (corresponding to compounds **3**, **1**, **2**, and **4**, resp.). Peak I was collected from 150 to 174 min, peak II from 186 to 200 min, peak III from 258 to 300 min, and peak IV from 330 to 345 min and were evaporated to yield 24.9, 50.1, 9.1, and 549.1 mg at 91.4, 94.5, 92.1, and 97.4% purity with recovery of 90, 94, 90, and 98%, respectively, as determined by HPLC. For collection of noneluted peak, V and VI in the first HSCCC, all fractions eluted after 350 min were combined and evaporated. The two-phase solvent system of HEMWat at a ratio of 3 : 7 : 5 : 5 was suitable for the separation of compounds **5** and **6**.  Figure 3 shows HSCCC separation of the EtOAc soluble fraction after HSCCC separation with the HEMWat (3 : 7 : 5 : 5, v/v) solvent system with *K* values of 2.46 and 1.24 for compounds **5** and **6**, respectively, and separated with good resolution. Peak V from 147 to 172 min and VI from 195 to 227 min were collected and concentrated. A total of 81.4 and 56.9 mg of compounds **5** and **6** were obtained at 96.4 and 98.2% purity with recovery of 94 and 95%. HPLC analysis of compounds **1**–**6** is shown in Figure 4.

### 3.4. Structural Determination and rAR Inhibitory Activity

The compounds were identified by comparing the LC-MS, UV, ^1^H-, and ^13^C-NMR to previously reported data. The compounds are protocatechuic acid (**1**), chlorogenic acid (**2**), caffeic acid (**3**), rosmarinic acid (**4**), luteolin (**5**), and methyl rosmarinic acid (**6**).

*Compound* ***1***. ^1^H-NMR (CD_3_OD, 400 MHz) *δ* 6.79 (1H, d, *J* = 8.0 Hz), *δ* 7.42 (1H, dd, *J* = 8.0 Hz and *J* = 2.0 Hz), *δ* 7.43 (1H, d, *J* = 2.0 Hz); ^13^C-NMR (CD_3_OD, 100 MHz) *δ* 168.15 (C-7), 115.99 (C-2), 117.38 (C-5), 122.47 (C-6), 122.47 (C-1), 145.73 (C-3), 150.85 (C-4); RT (retention time) 5.66 min, ESI-MS (*m*/*z*) 155 [M+H]^+^, MS-MS (*m*/*z*) 109 [M-COOH]^+^; UV (MeCN, *λ*_max⁡_ nm) 259 sh, 294. The ^1^H-NMR, ^13^C-NMR, MS, and UV data for compound **1** are identical to those reported previously [29, 30]. Compound **1** was identified as protocatechuic acid and did not exhibit inhibitory activity.

*Compound* ***2***. RT (retention time) 8.6 min, ESI-MS (*m*/*z*) 353 (M-H)^−^, 191 [quinic acid-H]^−^, 179 [caffeic acid-H]^−^; UV (MeCN, *λ*_max⁡_ nm) 298sh, 346 (max). The MS and UV data for compound **2** are identical to those reported previously in the literature [24]. Compound **2** was identified as chlorogenic acid and inhibited rAR in a concentration-dependent manner; its IC_50_ value was 3.16 *μ*M, which is similar to that of quercetin (5.07 *μ*M).

*Compound* ***3***. ^1^H-NMR (CD_3_OD, 400 MHz) *δ* 6.77 (1H, d, *J* = 8.1 Hz, H-5), *δ* 7.03 (1H, d, *J* = 2.0 Hz, H-2), *δ* 6.93 (1H, dd, *J* = 8.1 Hz and *J* = 2.0 Hz, H-6); ^13^C-NMR (CD_3_OD, 100 MHz) *δ* 171.46 (C-9), 115.96 (C-8), 147.22 (C-7), 116.91 (C-5), 115.51 (C-2), 123.27 (C-6), 128.23 (C-1), 147.45 (C-3), 149.87 (C-4); RT (retention time) 9.54 min, ESI-MS (*m*/*z*) 181 [M+H]^+^, MS-MS (*m*/*z*) 135 [M-COOH]^+^; UV (MeCN, *λ*_max⁡_ nm) 235 sh, 323. The ^1^H-NMR,^ 13^C-NMR, MS, and UV data for compound **3** are identical to those reported in the literature [31, 32]. Compound **3** was identified as caffeic acid, which showed weak inhibitory activity against rAR.

*Compound* ***4***. ^1^H-NMR (CD_3_OD, 400 MHz) *δ* 6.61 (dd, *J* = 1.9 Hz and *J* = 8.0 Hz), *δ* 6.69 (d, *J* = 8.0 Hz), *δ* 6.75 (d, *J* = 1.9 Hz), *δ* 6.77 (d, *J* = 8.1 Hz), *δ* 6.94 (dd, *J* = 8.1 Hz and *J* = 1.9 Hz), *δ* 7.04 (d, *J* = 1.9 Hz), *δ* 7.54 (d, *J* = 15.9 Hz), *δ* 6.26 (d, *J* = 15.9 Hz), *δ* 5.19 (dd, *J* = 4.3 Hz and *J* = 8.2 Hz), *δ* 3.00 (dd, *J* = 8.2 Hz and *J* = 14.2 Hz), *δ* 3.09 (dd, *J* = 4.3 Hz and *J* = 14.2 Hz); ^13^C-NMR (CD_3_OD, 100 MHz) *δ* 147.22 (C-4′), 150.15 (C-3′), 146.57 (C-3), 145.69 (C-4), 173.97 (C-9), 168.90 (C-9^'^), 148.16 (C-7′), 116.73 (C-8′), 75.06 (C-8); RT (retention time) 17.95 min, ESI-MS (*m*/*z*) 361 [M+H]^+^, MS-MS (*m*/*z*) 163 [M-COOH]^+^; UV (MeCN, *λ*_max⁡_ nm) 244,335 (max). The ^1^H-NMR, ^13^C-NMR, MS, and UV data for compound **4** are identical to those reported previously [25, 26, 33, 34]. Compound **4** was identified as rosmarinic acid. Compound **4** had the most potent rAR inhibitory activity (2.77 *μ*M).

*Compound* ***5***. RT (retention time) 22.27 min, ESI-MS (*m*/*z*) 287 [M]^+^, MS-MS (*m*/*z*) 153 [M-134]^+^, 86[M-201]^+^; UV (MeCN, *λ*_max⁡_nm) 253, 346 (max). The MS and UV data for compound **5** are identical to those reported previously [35]. Compound **5 **inhibited rAR in a concentration-dependent manner; its IC_50_ value was 6.34 *μ*M, similar to that of quercetin (5.07 *μ*M).

**Figure 5:**
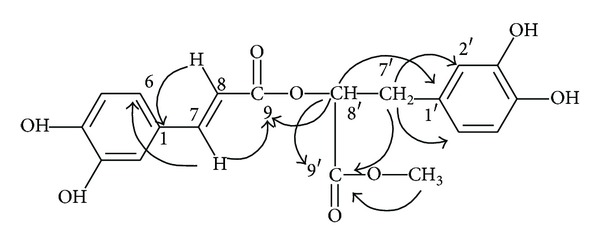
Main correlation HMBC for **6**.

*Compound* ***6***. ^1^H-NMR (CD_3_OD, 600 MHz) *δ* 6.57 (dd, *J* = 1.9 Hz and *J* = 8.0 Hz), *δ* 6.7 (d, *J* = 8.1 Hz), *δ* 6.71 (d, *J* = 1.7 Hz), *δ* 6.78 (d, *J* = 8.1 Hz), *δ* 6.96 (dd, *J* = 8.2 Hz and *J* = 1.5 Hz), *δ* 7.05 (d, *J* = 1.7 Hz), *δ* 7.55 (d, *J* = 15.8 Hz), *δ* 6.26 (d, *J* = 15.9 Hz), *δ* 5.19 (dd, *J* = 5.1 Hz and *J* = 7.6 Hz), *δ* 3.69 (s), *δ* 3.04 (dd, *J* = 8 Hz and *J* = 14 Hz), *δ* 3.09 (dd, *J* = 4.2 Hz and *J* = 14.2 Hz); ^13^C-NMR (CD_3_OD, 150 MHz) *δ* 126.20 (C1), 112.76 (C2), 145.43 (C3), 148.42 (C4), 114.92 (C5), 121.82 (C6), 146.56 (C7), 113.93 (C8), 166.93 (C9), 127.36 (C1′), 116.14 (C2′), 144.82(C3′),143.99 (C4′), 115.12 (C5′), 120.40 (C6′), 36.50 (C7′), 73.28 (C8′), 170.78 (C9′), 51.27 (C10′). RT (retention time) 23.82 min, ESI-MS (*m*/*z*) 374 [M]^+^, MS-MS (*m*/*z*) (% rel. int.) 163(100); UV (MeCN, *λ*_max⁡_ nm) 223 sh, 330. A cross peak between C-9 and the methyl proton was observed by a heteronuclear multiple bond connectivity (HMBC) experiment (Figure 5). The ^1^H-NMR, ^13^C-NMR, HMBC, MS, and UV data for compound **6** are identical to those reported previously [36]. Compound **6** was identified as methyl rosmarinic acid. Compound **6** inhibited rAR in a concentration-dependent manner; its IC_50_ was 4.03 *μ*M, significantly lower than that of quercetin (5.07 *μ*M).

## 4. Conclusions

Crude extracts of the aerial portion of *P. frutescens* obtained by extraction with 99.5% methanol showed considerable rAR inhibitory activity. The EtOAc soluble fraction of the crude extract exhibited remarkable inhibitory activity against rAR (IC_50_ = 1.92 *μ*g/mL). Therefore, an investigation of inhibitory compounds from an EtOAc soluble fraction of the crude extract was followed by an effort to identify the inhibitory compounds by combining HPLC microfractionation with a 96-well enzyme assay. The rAR inhibitory activity profile showed that peaks **2** and **4 **exhibit potent inhibitory activity. Structural analyses of these peaks were then carried out by LC-MS^n^. The inhibitory compounds were tentatively identified as chlorogenic acid and rosmarinic acid. Furthermore, in a search for additional rAR inhibiting compounds, 6 pure compounds with different levels of rAR inhibitory activity were isolated from an EtOAc soluble fraction by HSCCC and identified as protocatechuic acid (**1**), chlorogenic acid (**2**), caffeic acid (**3**), rosmarinic acid (**4**), luteolin (**5**), and methyl rosmarinic acid (**6**). Specifically, rosmarinic acid exhibited the most potent AR inhibitory activity with an IC_50_ of 2.77 *μ*M (Table 3). The HPLC microfractionation plus enzyme assay system generated biological and chemical information and provided a valuable tool for screening and identification of rAR inhibitors in complex samples.

## Figures and Tables

**Table 1:** Inhibitory effects of the crude extracts and fractions of *P. frutescens* on rAR.

Sample	IC_50_ (*μ*g/mL)^a^
MeOH extract	9.34
*n*-Hexane fr.	—
CH_2_Cl_2_ fr.	9.06
EtOAc fr.	1.92
*n-*Butanol fr.	2.89
Water fr.	—
Quercetin^b^	0.77

^a^The IC_50_ value was defined as the concentration at 50% inhibition.

^b^Quercetin, positive control.

**Table 2:** Partition coefficient *K*-value of target compounds **1**–**6** in different two-phase solvent systems used in HSCCC.

*n*-Hexane	EtOAc	MeOH	ACN	Water	**1**	**2**	**3**	**4**	**5**	**6**	Settling time (s)
1.5	5	1.5	4.5		0.93	0.95	0.79	1.41			32
1.5	5	1.5		4	1.01	1.24	0.12	1.49	>2.5		12
1.5	5	1		5	1.26	1.86	1.17	2.43			18
2	8	4		6					4.22	2.6	27
3	7	3.5		6.5					4.51	2.31	28
3	7	5		5					2.46	1.24	18

**Table 3:** Inhibitory effects of compounds **1**–**6** isolated from EtOAc-soluble fractions of *P. frutescens* extract on rAR.

Number	Compound	IC_50_ (*μ*M)^a^
**1**	Protocatechuic acid	—
**2**	Chlorogenic acid	3.16
**3**	Caffeic acid	—
**4**	Rosmarinic acid	2.77
**5**	Luteolin	6.34
**6**	Methyl rosmarinic acid	4.03
	Quercetin^b^	5.07

^a^The IC_50_ value was defined as the concentration at 50% inhibition.

^b^Quercetin, positive control.
